# Innate Immune Invisible Ultrasmall Gold Nanoparticles—Framework for Synthesis and Evaluation

**DOI:** 10.1021/acsami.1c02834

**Published:** 2021-05-12

**Authors:** Geyunjian Harry Zhu, Mohammad Azharuddin, Rakibul Islam, Hassan Rahmoune, Suryyani Deb, Upasona Kanji, Jyotirmoy Das, Johannes Osterrieth, Parminder Aulakh, Hashi Ibrahim-Hashi, Raghav Manchanda, Per H. Nilsson, Tom Eirik Mollnes, Maitreyee Bhattacharyya, Mohammad M. Islam, Jorma Hinkula, Nigel K. H. Slater, Hirak K. Patra

**Affiliations:** † Department of Chemical Engineering and Biotechnology, University of Cambridge, Cambridge CB3 0AS, U.K.; ‡ Department of Biomedical and Clinical Sciences (BKV), Linkoping University, Linkoping 581 83, Sweden; § Department of Immunology, Oslo University Hospital, University of Oslo, Oslo 0372, Norway; ∥ Department of Biotechnology, Maulana Abul Kalam Azad University of Technology (MAKAUT), Kolkata 700064, India; ⊥ Institute for Manufacturing (IfM), University of Cambridge, Cambridge CB3 0FS, U.K.; # Linnaeus Center for Biomaterials Chemistry, Linnaeus University, Kalmar 391 82, Sweden; ¶ Research Laboratory, Nordland Hospital, Bodø, and Faculty of Health Sciences, K.G. Jebsen TREC, University of Tromsø, Tromsø 9037, Norway; ∇ Institute of Haematology and Transfusion Medicine, Calcutta Medical College, Calcutta 700073, India; ○ Massachusetts Eye and Ear and Schepens Eye Research Institute, Dept of Ophthalmology, Harvard Medical School, Boston, Massachusetts 02114, United States; ⧫ Department of Surgical Biotechnology, University College London (UCL), London NW3 2PF, U.K.

**Keywords:** ultrasmall nanoparticles, process engineering, immunocompatibility, complement-safe, coagulation-safe, pro-inflammatory cytokine, biocompatibility

## Abstract

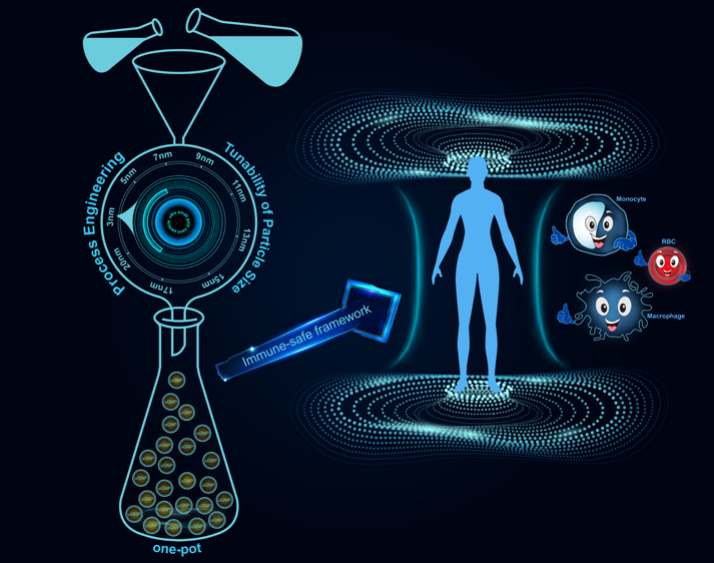

Nanomedicine is seen as a potential central player in the delivery of personalized medicine. Biocompatibility issues of nanoparticles have largely been resolved over the past decade. Despite their tremendous progress, less than 1% of applied nanosystems can hit their intended target location, such as a solid tumor, and this remains an obstacle to their full ability and potential with a high translational value. Therefore, achieving immune-tolerable, blood-compatible, and biofriendly nanoparticles remains an unmet need. The translational success of nanoformulations from bench to bedside involves a thorough assessment of their design, compatibility beyond cytotoxicity such as immune toxicity, blood compatibility, and immune-mediated destruction/rejection/clearance profile. Here, we report a one-pot process-engineered synthesis of ultrasmall gold nanoparticles (uGNPs) suitable for better body and renal clearance delivery of their payloads. We have obtained uGNP sizes of as low as 3 nm and have engineered the synthesis to allow them to be accurately sized (almost nanometer by nanometer). The synthesized uGNPs are biocompatible and can easily be functionalized to carry drugs, peptides, antibodies, and other therapeutic molecules. We have performed *in vitro* cell viability assays, immunotoxicity assays, inflammatory cytokine analysis, a complement activation study, and blood coagulation studies with the uGNPs to confirm their safety. These can help to set up a long-term safety-benefit framework of experimentation to reveal whether any designed nanoparticles are immune-tolerable and can be used as payload carriers for next-generation vaccines, chemotherapeutic drugs, and theranostic agents with better body clearance ability and deep tissue penetration.

## Introduction

Nanotechnology has had a huge influence in the field of biomedicine, such as in therapy, diagnosis, vaccination, and biosensing.^1^ Advancements in the field have allowed scientists and engineers to create novel therapeutic strategies and formulations to achieve better efficacy, improve safety, enhance targeting, and reduce systemic toxicity. Nanomedicine has also been viewed as a leading element in personalized medicine in the future.^2,3^ Although nanomaterials are already met in our daily life,^4,5^ the safety concern of nanosized biomaterials, especially non-biodegradable nanoparticles, remains a hurdle for their wider application.^6^ Nanomaterials, having sizes from 1 to 100 nm, can have unique interactions with biological components and can overcome biological barriers due to their nanometric size, which can be both beneficial and potentially harmful. One of the major safety concerns of using nanoparticles is the long-term toxicity when these materials are not effectively eliminated from the body.^6^ Prolonged and absorbed nanomaterials can potentially lead to protein denaturation,^7,8^ cellular apoptosis,^9^ activation of platelets,^10^ and DNA damage upon their entry into organelles such as mitochondria and the nuclei.^11,12^ The two major clearance pathways of nanomaterials are renal and hepatic pathways, with the general size cutoff being 6–8 nm, based on the fenestrae size in the glomerular basement.^13^ The smaller nanoparticles that can go through the renal outlet are usually cleared quickly, while larger nanomaterials that accumulate in the liver and spleen are metabolized over a longer period of time.^14^ Controllable and effective renal clearance is a more preferable elimination route than the hepatobiliary metabolism.^15^ Therefore, an ultrasmall size (sub-10 nm) is highly desirable for efficient nanomaterial elimination after their intended action.

The interactions between nanoparticles and the immune system have been subjected to extensive investigation recently as an effort toward translational research.^16−19^ This has been studied from two perspectives, namely, the effects of nanoparticles on immune system functions and the disruptions of immune systems on nanomedicine efficiency. Nanomaterials can not only have adverse effects on the normal function of the immune system but also elicit undesirable immune responses (e.g., by monocytes and macrophages). Macrophages upon exposure to foreign nanoparticles may release pro-inflammatory cytokines that could enhance, suppress, or skew the immune response. Therefore, it is essential to assess immune compatibility of nanosystems for biomedical applications. Secondly and equally importantly, the interaction with the immune system also largely determines the efficiency of nanoparticles as the uptake by immune cells can lead to premature clearance of the nanoparticles. In order to achieve the desired clinical effect, nanocarriers have to be injected intravenously, with an eye to reach specific and targeted drug delivery.^20,21^ In an *in vivo* situation, they are exposed to confront with immune cells,^22,23^ and this leads to the generation of cascade reactions that renders the nanomaterials ineffective and will ultimately be cleared from the body (including clearance through complement activation). Intravenously administered nanomaterials encounter a huge protein load when it enters the blood stream. A phenomenon, termed “opsonization”, takes place when a group of proteins called opsonins get adsorbed onto the surface of nanomaterials.^20,22^ In general, opsonization determines the fate of the nanoparticles inside the body, which predominantly depends on the amount and type of proteins adsorbed onto the particle’s surface.^7,20,22−24^ Serum albumin readily adsorbed onto the surface of nanoparticles, followed by a cascade of reactions, such as binding or displacement of the adsorbed albumin with fibrinogen. The immune system is activated on recognizing the adsorbed protein on the nanoparticle, and this in turn causes activation of the complement cascade.^22,23^

Complement activation involves binding of recognition proteins that can trigger further activation of the complement cascade. Cleavage of C3 is a key event, which ultimately opsonizes the nanoparticle with C3b for phagocytosis.^22^ Nanoparticles with low protein binding capacity, which can remain longer in the blood circulation, are desired since they have low potential to activate the complement system.^23^ The response of nanoparticles on the activation of the complement system is difficult to study *in vivo*, and hence, performing *in vitro* tests beforehand is beneficial for clinical translation. The interaction of proteins onto the nanoparticle surface can also influence blood coagulation.^25^ Blood clotting is a process leading to the activation of thrombin for cleavage of fibrinogen for the formation of a fibrin network.^26^ The coagulation cascade can be activated via two pathways: (i) the intrinsic-determined by activated partial thromboplastin time (APTT) and the (ii) extrinsic-estimated by prothrombin time (PT). Both screening tests measure the approximate time it takes for blood to clot. A blood-compatible coagulation-safe nanoparticle is expected to have no effect on the coagulation time. A shorter coagulation time could lead to blood clotting risks by the formation of a thrombus, while a longer coagulation time would cause hemorrhage.^23^

To achieve maximum efficacy and low toxicity, intense research has been focused on modulating the physiochemical properties of nanoparticles, such as size and surface chemistry.^26^ In general, smaller-size nanoparticles are more stable in the colloidal form and therefore more favorable due to their higher stability, and they can efficiently avoid detection by the immune system and subsequent clearance.^21,28^ Smaller-size nanoparticles have also led to better tumor penetration,^29^ less macrophage uptake,^30^ a longer blood circulation time,^31^ and higher renal clearance.^15^ A larger specific surface area and many other unique physical properties^32^ are only possible when the particle size reaches sub-10 nm and are extremely useful for diagnostic purposes.^33^ The nanoparticle size is determined by both the core and the thickness of coating and collectively dictates the properties of the particles. Nanoparticles with a smaller core and larger PEG coatings confer a longer blood circulation time than a similar size but composed of a bigger core and thinner coatings.^34^ Glutathione-coated gold nanoparticles (GNPs) undergo faster renal clearance than those coated with BSA and heavily accumulate in the liver and spleen in mice.^35^ Therefore, a small core is a prerequisite for rendering small particles. Although it appears that smaller particles should be beneficial in biomedical applications, an optimal particle size is believed to be application-specific.^34^ Therefore, the abilities to fabricate ultrasmall nanoparticles and to finely tune their size are both highly desirable. Among all types of materials, GNPs have taken the center stage of nanoparticle-based biomedical applications for years owing to the significant development of their method of synthesis, diverse surface modifications, and excellent physical properties.^36^ Countless fabrication methods for making GNPs with a wide range of sizes and geometries have been reported since Faraday discovered the very first GNP synthesis method via the reduction of tetrachloroaurate with phosphorus.^37^ One of the most frequently used methods for GNP synthesis is the wet chemical reduction of tetrachloroauric acid (HAuCl_4_) using sodium citrate (NaCit) pioneered by Turkevich.^38^ However, the Turkevich method fails to prepare sub-10 nm ultrasmall GNPs (uGNPs) or to tune the particle size very precisely. To prepare uGNPs, strong reducing agents, such as NaBH_4_, are usually used with strong capping ligands to limit particle growth.^39^ The use of strong and often toxic capping agents makes it difficult for future surface modifications and requires detoxification steps for use in biological settings apart from the huge polydispersity.^40^ Mühlpfordt, and later Slot and Geuze, proposed that the use of tannic acid (TA) and NaCit as combined reducing agents led to the synthesis of sub-10 nm uGNPs.^41,42^ However, the reaction mechanism of this TA–NaCit method is poorly understood, and although the method has some tunability over particle size, it does not show systematic control at the nanometer-by-nanometer level.

In the current report, a series of uGNPs with sizes ranging from 3 to 15 nm were fabricated with an engineered synthesis method that comprised an initial seed formation step and multiple discrete growth steps to achieve nanometer-by-nanometer tunability. The initial seeding reaction was achieved with the TA–NaCit method for which a careful kinetic study was conducted to provide new insight into the reaction mechanism. The TA–NaCit method was then utilized as the seed formation step, with the help of recent advances in the seeded growth method,^43,44^ to devise a process-engineered synthesis method for uGNPs with a finely controlled size for biomedical applications. *In vitro* and *ex vivo* studies were conducted to demonstrate the immune tolerability and blood compatibility of the synthesized uGNPs. While the synthetic platform was extremely valuable for making immune-safe and precisely tunable nanoparticles to meet the specific size requirements in any biomedical applications, the framework of testing their immune compatibility can be beneficial to a wider collection of nanomaterials for biomedical applications.

## Results and Discussion

The first objective was to develop a process-engineered synthesis procedure to obtain sub-10 nm uGNPs with a precisely tuned particle size for biomedical applications. The synthesis process was created based on the concept of controlled seed-mediated growth with suppressed secondary nucleation.^43^ The initial seed formation was based on standard TA–NaCit reaction conditions reported earlier, including reaction temperature, pH, and a reagent addition sequence.^45−48^ Additionally, a controlled reducing environment was created by pre-boiling the reaction solution and implementing a continuous nitrogen purge throughout the reaction to exclude as much oxygen.

**Figure 1:**
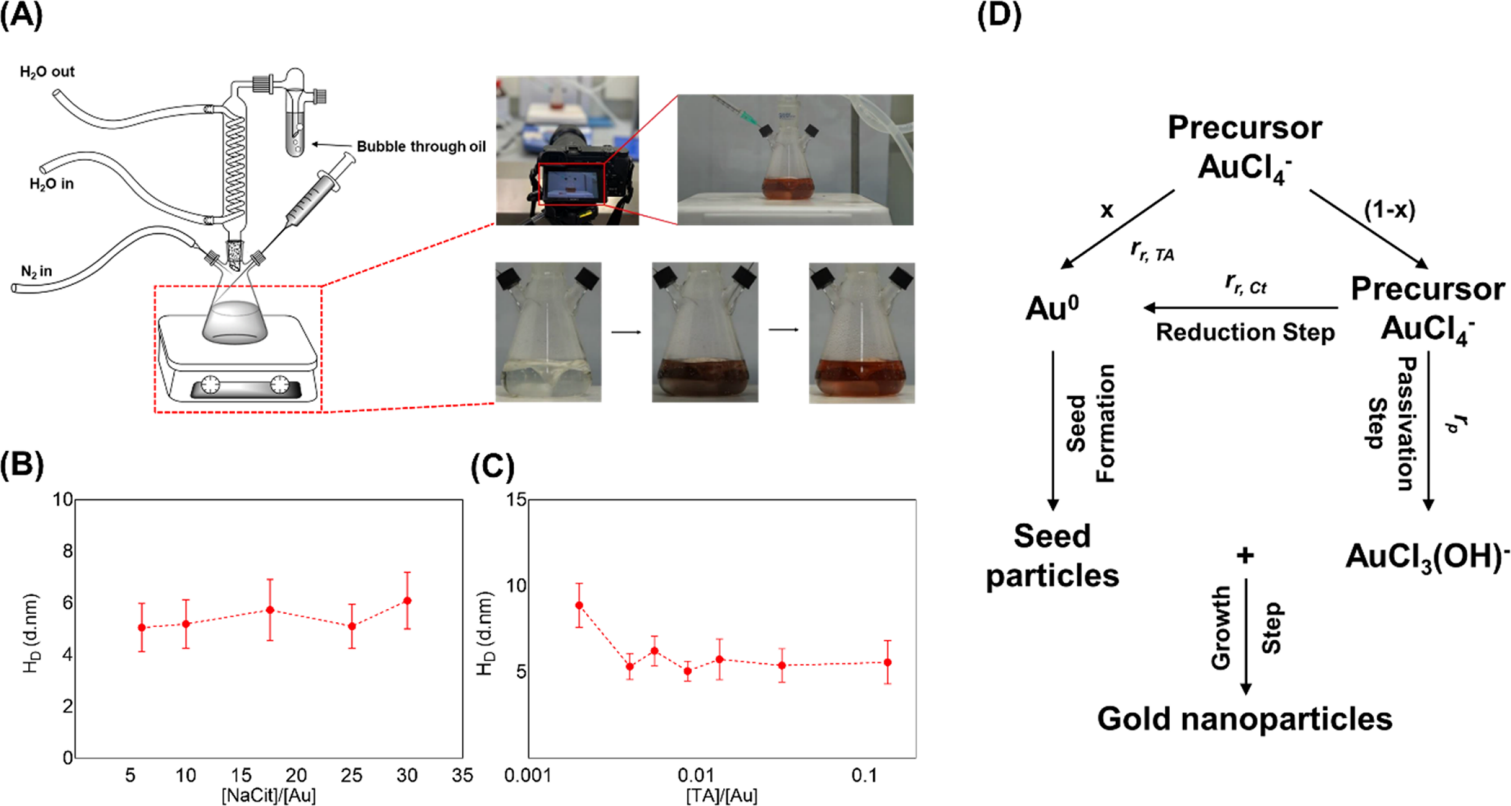
(A) One-pot synthesis setup for seeded growth of uGNPs. (B,C) Particle size change as functions of NaCit and TA to gold precursor ratios using the TA–NaCit method. (D) Proposed mechanism of GNP formation by the TA + NaCit method.

In an effort to better understand the reaction mechanism and study the factors affecting the size of particles, the role of TA and NaCit as reducing agents on the reaction rate and the final particle size of uGNPs was thoroughly investigated. The real-time plasmon color of the reaction colloid was analyzed with the RGB (red, green, and blue) model, and the relative intensity of the red component was taken as the reaction progression.^49^ Reduction of the precursor was found to be the first reaction in the seed-mediated synthesis using the citrate synthesis method as well as the combination of TA and NaCit.^44,50^ This suggested that addition of TA had a very significant role in accelerating the reaction rates and might play a dominant role in the reduction of the precursor. Piella et al. found that the kinetics is largely regulated by the concentration of TA, while NaCit controls the growth process of the initial seeds.^44^ Thus, the changes of both TA/Au and NaCit/Au ratios were investigated, and both showed the impact on the final particle size as demonstrated in Figure 1B,C. Particle size is relatively large at low TA concentrations and reaches a plateau as TA concentration increases. NaCit on the other hand appears to have a smaller impact on the size but does change the reaction kinetics.

The role of TA and NaCit in the initial reduction was explored further by checking the reaction profiles as a function of time at different ratios of NaCit/Au while fixing the TA concentration and varying the TA/Au ratio at a constant NaCit concentration. The increase in the NaCit–Au ratio expedites the reaction, thereby leading to faster attainment of final sizes of uGNPs, while the initial reaction rate was unaffected with the TA holding constant (Figure S1A). It was found that the increase in TA/Au led to an increase in the initial reaction rate as exhibited by the change of the plasmonic red component (Figure S1B). This indicated that both NaCit and TA might play a joint role in the reduction; however, TA is the dominant reducing agent. The relationship between reducing agents and initial reduction rate was further investigated (Figure S2). The initial reaction rate was calculated as follows:
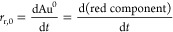
1

NaCit undergoes speciation in the solution forming Ct^3–^, CtH^2–^, CtH_2_^–^, CtH_3_, or a combination thereof. Ojea-Jiménez and Campanera^51^ reported that the reducing agent in the case of sodium citrate-mediated synthesis was CtH_2_^–^, while Kettemann et al.^52^ and Agunloye et al.^50^ found that it was CtH^2–^. The concentrations of CtH^2–^ and CtH_2_^–^ were calculated as described in a previous report,^60^ and both the species were tested. TA-mediated reduction of Au^3+^ ions to form Au^0^ involves each phenolic group of TA donating two electrons, thereby forming quinone; TA was thus assumed to be the reacting species. The rate equation used was as follows:
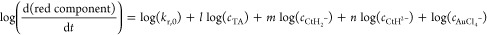
2

TA was found to be the dominant reducing agent (Figure S2). The plots of reaction rate as a function of CtH_2_^–^ and CtH^2–^ are provided in the Supporting Information (Figure S1). A linear correlation between log(*r*_r,0_) and log(*C*_TA_) is obtained, and the value of l is estimated to be 1.1. Equation 2 can thus be rewritten as follows:
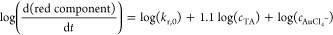
3
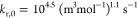


Agunloye et al.^50^ presented a reaction scheme for the formation of GNPs from the precursor in the citrate synthesis method. In the case of TA + NaCit-mediated synthesis, TA was found to be the main reducing agent; however, sodium citrate also led to reduction, although at a much lower rate than TA. Based on the evidence set out above, the reaction mechanism of TA + NaCit-mediated GNPs is proposed (Figure 1D). Reduction occurs via either the TA route or the sodium citrate route, and the fraction of the gold precursor undergoes TA is assigned as *x* and the remaining is therefore assigned as 1 – *x*. The reduced precursor undergoes seed formation, and finally, the seeds and the passivated precursor [AuCl_3_(OH)^−^] grow to form GNPs. This is supported by Figure S3, which suggests that other than the initial “jump” in the reaction profile, Mühlpfordt’s method and the Turkevich synthesis are identical. To test the proposed mechanism, the reduction and passivation reactions were modelled. Previously reported reaction kinetics was used for citrate-based reduction and passivation steps.^50^ CtH_2_^–^ was assumed to be the species responsible for citrate-based reduction,^50^ and [OH^–^] was assumed to remain constant throughout. If each polyphenolic group were able to donate 2 electrons, forming quinones in the process, each TA molecule can provide 20 electrons.^44^ Three electrons are required to reduce Au^3+^ to Au^0^. Therefore, for modelling TA consumption, a 3/20 stoichiometric ratio was used, giving the following rate equations:
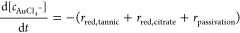
4
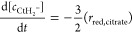
5
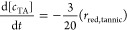
6
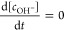
7
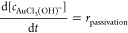
8

Simulated plots of AuCl_4_^–^ and TA as functions of time are shown in Figure S4. Varying the NaCit/Au ratio from 6 to 25, while keeping TA constant, did not show any effect on the AuCl_4_^–^ profile. The initial sharp rise in the absorbance in Figure S3B was attributed to the TA-based reduction which is evident from Figure S4B since TA is consumed very fast for all NaCit/Au ratios. Figure S5 shows that as TA/Au increases the initial jump becomes steeper and more pronounced, in line with the mechanism presented in Figure 1D; more TA results in more precursor being used up in the initial jump.

Having defined a mechanism in Figure 1D, an expression for the final diameter of nanoparticles as a function of seed diameter, TA, and citrate concentrations was proposed. Based on a previous report,^50^
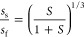
9

Following Agunloye et al.,^50^ the selectivity is defined as the ratio of reduction to passivation as follows:
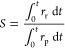
10

However, the rate of reduction is a summation of rate of reduction due to TA and citrate, so the equation now can be written as follows:
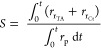
11
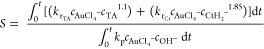
12

Assuming that the values of *c*_CtH2^–^_, *c*_TA_, and *c*_OH^–^_ do not change significantly from their values at quasi-equilibrium, denoted as *c*_CtH2^–^,0_, *c*_TA,0_, and *c*_OH_^–^_,0_, respectively, we can write
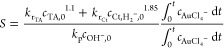
13
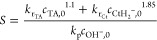
14

Equations 9 and 14 can be used to calculate the final size of nanoparticles if the seed diameter is known. The proposed mechanism forms the basis of deriving a complete model for the formation of GNPs using TA + citrate mediated synthesis which shall form part of the future work.

**Figure 2:**
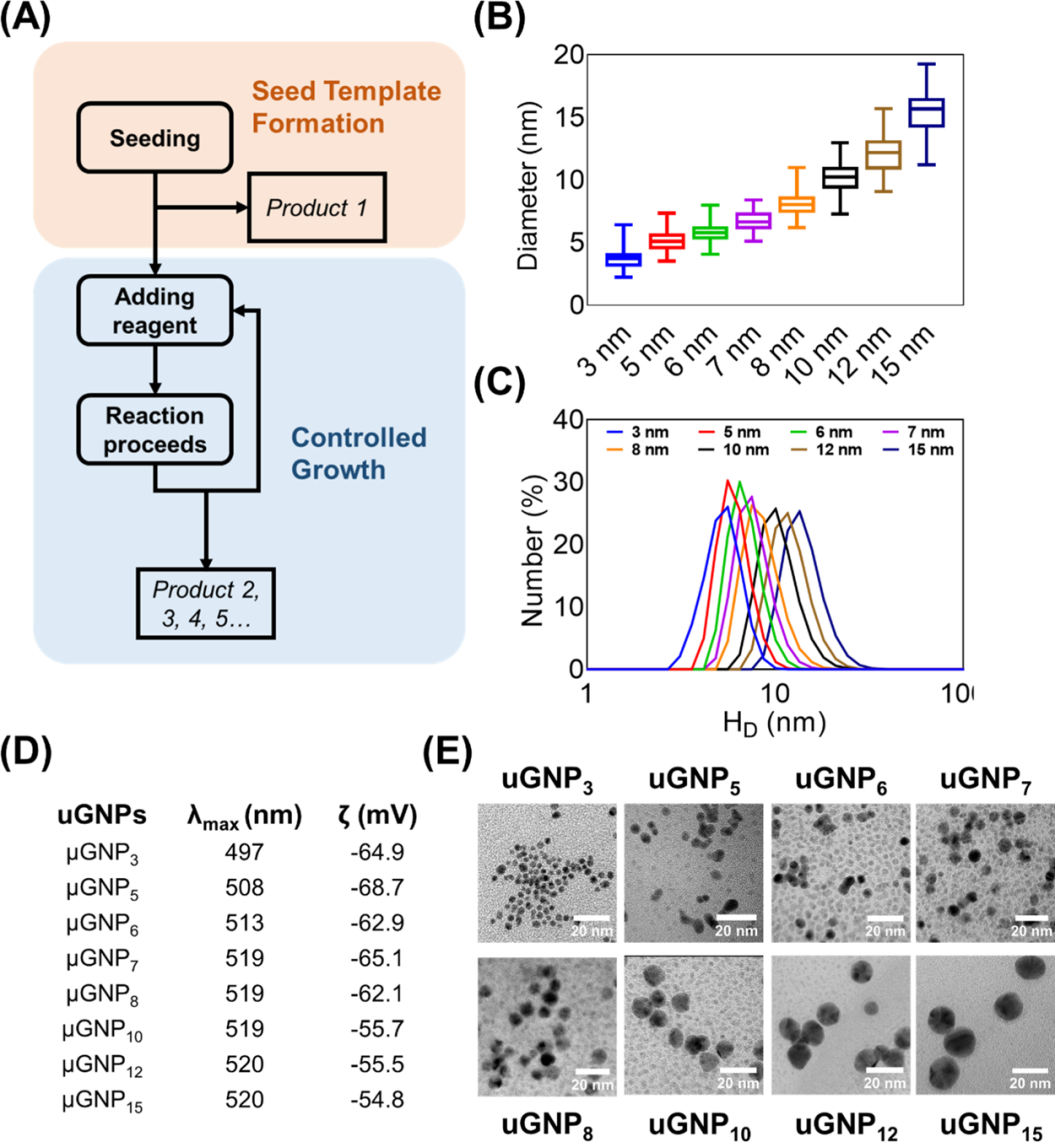
Synthesis and characterization of uGNP series. (A) Flowchart showing the process of uGNP synthesis consisting of the seed formation step and the multiple controlled growth step. Particle size distribution obtained with (B)TEM imaging and (C) DLS. (D) LSPR and ζ-potential as measured by UV–vis spectrometry and ELS. (E) TEM image gallery for uGNPs.

With better understanding of the kinetics and mechanism of the TA–NaCit method, it was used to synthesise small uGNP seeds which were then taken as templates for further seed-mediated growth reactions to obtain a series of uGNPS. The uGNPs are characterized for their sizes, geometry, localized surface plasmon resonance (LSPR), and ζ-potentials using dynamic light scattering (DLS), transmission electron microscopy (TEM), UV–vis spectroscopy, and electrophoretic light scattering (ELS) (Figure 2). A gradual increase from ∼3 to ∼15 nm is observed with progression of the synthesis. TEM imaging was also performed to confirm the size and shape of the particles. As expected, there is a size difference observed due to the hydrodynamic diameter measured by DLS and the bare size by TEM (Figure 2B,C). In general, for smaller particles, their hydrodynamic diameters (*H*_D_) obtained from DLS are about 1 nm larger than the diameter assessed by TEM. The ζ-potentials of freshly prepared uGNPs were also measured (Figure 2D). Large negative ζ-potentials (∼−60 mV) are observed for all uGNPs with different sizes. The negative surface charge is due to the citrate groups, and the large absolute ζ-potential values indicate high colloidal stability. The LSPR is recorded for as-synthesized uGNPs (Figures 2D and S6). The maximum absorption wavelength (λ_max_) in UV–vis spectra shifts from 497 to 520 nm for uGNP_3_ to uGNP_15_, which validates that larger particles are successfully obtained as the reaction progresses.

The theoretical particle size of each uGNP sample was calculated (Figure S8) based on the measured seed size and the amount of the Au precursor injected at each reaction step, assuming 100% yield. The results showed that the theoretically calculated particle sizes fall well within the standard deviation of measured particle sizes (Figure S8). Notably, the theoretical particle sizes are slightly larger than the measured particle size. This could likely be due to the fact that the extent of reaction is slightly lower than 100% and due to the loss of material during experimental operation. The stability of as-prepared citrate capped uGNPs in phosphate-buffered saline (PBS), fetal bovine serum (FBS) solution, and the culture medium was studied by incubating uGNPs in different media and measuring the change in UV–vis spectra (Figure S9). The uGNPs immediately started to aggregate in PBS as the color of the solution changed from red to purple, and later, a visible solid precipitate emerged. In contrast, uGNPs stayed relatively stable in both FBS and the culture medium for at least 24 h. This could be due to the adsorption of serum protein and other amino acids onto the uGNP surface helping to stabilize the particles.

In nanomedicine, coating the nanosurface with an antifouling molecule that can resist unwanted interactions with blood components, imparting “stealth” properties, is a common practice. PEGylation with PEG is the most extensively used “stealth” in biomedical applications due to its widespread research on safety in humans and classified as generally regarded as safe by the FDA. PEG is used as a stabilizing and protective agent in several clinically approved nanoformulations (e.g., Doxil, Oncaspar, and Onivyde)^53^ and thus remains the gold standard for nanoparticle coating. To demonstrate the ease of functionalization potential of uGNPs, 8-arm PEG-SH (MW = 10 kDa) was used to coat uGNPs. The gold-thiol linkage was widely used for gold surface modification to form a self-assembled monolayer.^54^ The PEGylation reaction in this study was easily conducted by simple incubation of 8-arm PEG-SH with as-prepared uGNPs at an elevated temperature for 1 h. The minimum PEG/uGNP ratios needed to cover the uGNPs was studied (Figure S10). It was estimated that ∼3.3 PEG molecules per nm^2^ (stoichiometry ratio) are needed to fully cover the uGNP surface. Similar trends were also observed with UV–vis and ζ-potential measurements.

**Figure 3:**
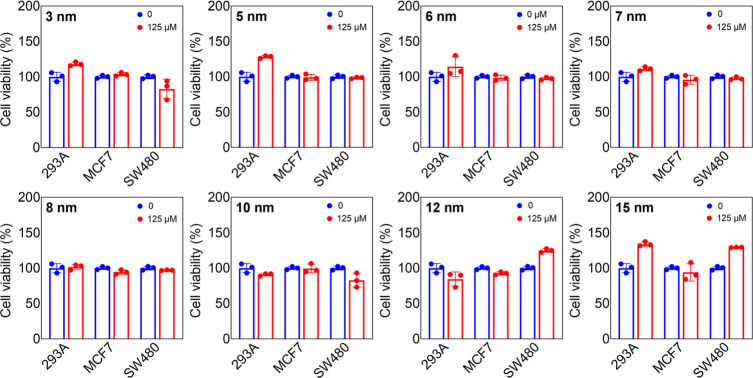
*In vitro* cytotoxicity effect of uGNPs of all eight sizes on three different cell lines (293A, MCF7, and SW480) at 72 h and at the highest dosage. Data are expressed as the mean ± SD.

Although the cytotoxicity of GNPs has been extensively reported in the literature, no conclusive evidence on their biosafety and biocompatibility has been presented.^27,55^ The *in vitro* cytotoxicity profile of uGNPs was assessed in human embryonic kidney cells (293A), breast cancer cells (MCF7), colon adenocarcinoma cells (SW480), peripheral blood monocyte cells (THP-1), and THP-1 differentiated macrophage-like cells after exposure for 24, 48, and 72 h, respectively. To estimate the biocompatibility of uGNP, we have cross-examined with two assays with dissimilar mechanisms for cell death: one with an intracellular indicator and another with an extracellular indicator. The 3-(4,5-dimethylthiazol-2-yl)-5-(3-carboxymethoxyphenyl)-2-(4-sulfophenyl)-2*H*-tetrazolium (MTS) assay was employed for cell viability by determining the activity of the mitochondrial dehydrogenase. The cell death was determined using a lactate dehydrogenase (LDH) assay that measures the leaked cytoplasmic contents by cells with compromised cell membranes. In parallel, the MTS proliferation assay is used to evaluate the cytotoxicity of all uGNPs with three different cell lines, 293A, MCF7, and SW480 at 24, 48, and 72 h at three different dosages (Figures 3 and S11). In general, uGNPs of all sizes exhibit very low cytotoxicity for all three cell lines. Interestingly, in some cases of 293A (3, 5, 6, and 15 nm) and SW480 (12 and 15 nm) cell lines, some promotion of proliferation is observed like few previous reports.^27^ The results confirm that uGNPs do not have any acute cytotoxic effects on multiple cell types.

**Figure 4:**
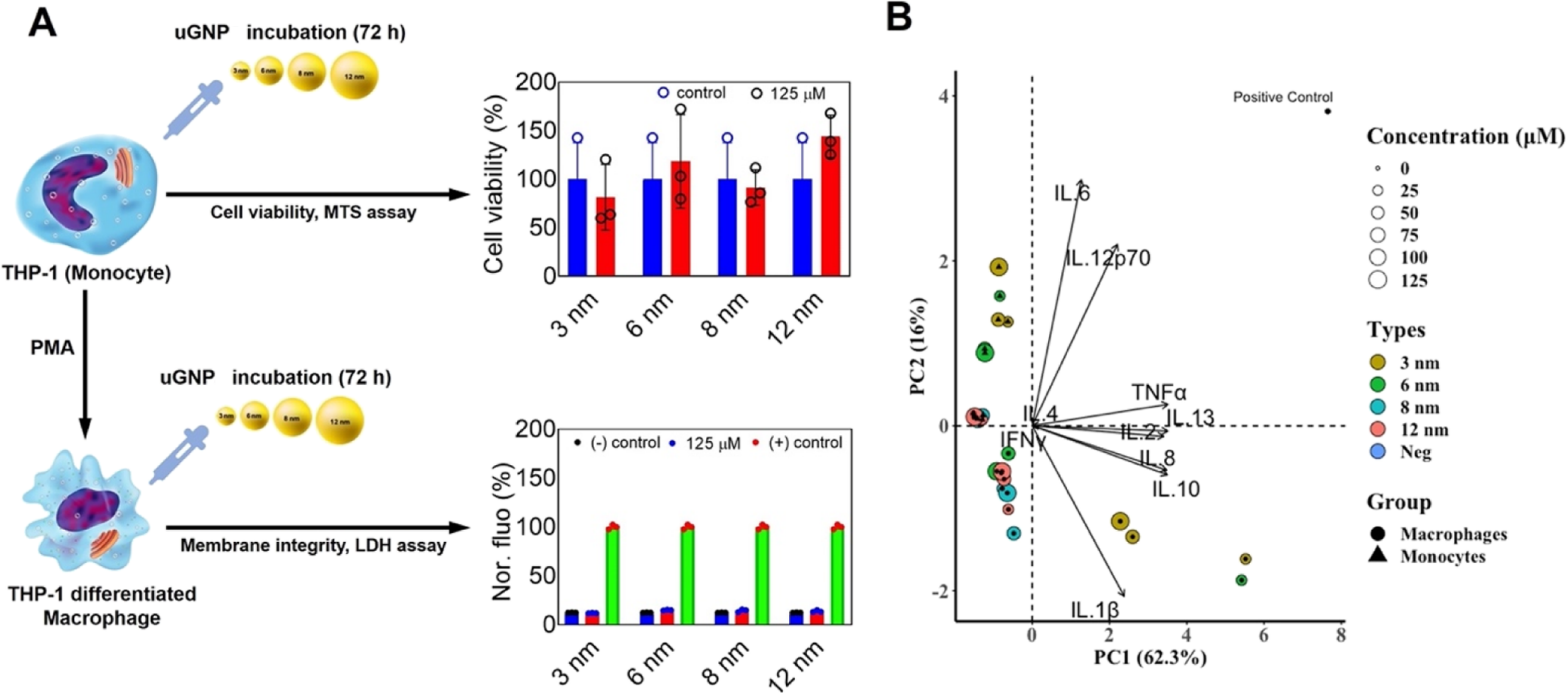
(A) Effect of uGNPs on undifferentiated, MTS assay (upper) and differentiated THP-1, LDH assay (lower) cell lines over a 72 h period and at the highest uGNP dosage. Data are expressed as the mean ± SD. ***p < 0.001 and *p < 0.02 compared to the untreated/control. (B) Cytokines and uGNPs illustrating sources of variance in the data following PCA. Monocytes (solid triangles) were grouped together along with the PC2 and macrophages (solid circles) were dispersed in both dimensions. Different uGNPs were marked with different colors (uGNP3: brown, uGNP6: green, uGNP8: cyan, and uGNP12: red), and their concentration scale is represented with a circle size (a lower concentration to a higher concentration defines as a smaller circle to a larger circle). In the biplot, different cytokines (variances) were displayed on the positive axis of PC1, representing their effective tendency on the data. IL-4 and IFNγ showed no variability within the data set, whereas IL-1β, IL-2, IL-8, IL-10, and IL-13 were placed in the same quadrant with uGNP3 (all concentrations) and uGNP6 (the lowest concentration). IL-6 and IL-12p70 were lying outside of this quadrant and in the same direction with the positive control sample (navy blue color). PC2 portrayed the separation of cell types (monocytes and macrophages).

As mentioned, an *in vitro* study of nanoparticle interactions with the immune system is crucial to indicate a concentration dosing threshold that could be used to commence *in vivo* studies. The cytotoxicity of uGNPs (3, 6, 8, and 12 nm) was also assessed in THP-1 and THP-1 differentiated macrophages using the MTS and LDH assays over an incubation window of 72 h. Even after exposure at a high dose (125 μM), both macrophage and monocyte-like THP-1 cells showed no significant reduction in viability when compared to the control or untreated cells (Figure 4A). The cell membrane integrity assay on THP-1 macrophages exhibited no observable leakage of cytoplasmic contents into the cell culture medium as represented in Figure 4A. These results suggest that uGNPs are highly compatible and do not impair phorbol-12-myristate 13-acetate (PMA)-induced THP-1 differentiated macrophage processes *in vitro*. The results add insight with the view of using these ultrasmall particles in drug delivery as well as being consistent with other studies previously reported in macrophages and human leukemia cells.^17,56^ The data obtained here during the course of this study showed that there were no significant effects on the viability of different cell lines even after 72 h exposure to increasing doses of uGNPs. These results are in agreement with previously published reports.^17,57^ Also, the shape and surface charge of uGNPs play a pivotal role in determining their biocompatibility. The as-synthesised uGNPs being spherical and negatively charged display lesser toxicity as compared to rod-shaped and positively charged ones.^58,59^ Further *in vivo* studies need to be carried out in order to fully elucidate the biodistribution and potential adverse responses of uGNPs. Figure 4B shows the principal component analysis (PCA) plot for the uGNPs and pro-inflammatory cytokines. Interestingly, all cytokines were placed only in the positive direction of PC1 with two different clusters. IL-2, IL-8, IL-10, and IL-1β were positioned along with uGNP_3_ and uGNP_6_ in the negative quadrant of PC2, whereas IL-6 and IL-12p70 were separately clustered in the positive quadrant of PC2. IL-4 and IFNγ showed no profound effects on the data.

Structured screening processes of immunotoxicity are one of the desires to be implemented for environmental and pharmaceutical products,^60^ and nanoparticles should be included as well while considering biomedical applications. For nanomaterials to be used *in vivo*, it is crucial to check their immuno compatibility. Especially for vaccination applications, the immunogenicity of the nanomaterials has a direct impact on the success in eliciting desired immune response. Whether the complement activation induced by uGNPs, were investigated specifically. In general, the complement system acts in immune surveillance by instantly responding to eliminate the pathogens, other foreign substances, or damaged substrates.^61^ Different signaling pathways involve different proteins that can trigger activation of the complement cascade, and the protein C3 specifically acts when the above cascade is activated. Actuation of C3 is a key event, which ultimately eliminates the nanoparticles that trigger the whole process.^22^ Nanoparticles with low protein binding capacity are generally desired since they have a lower potential to activate the complement system and thus can remain longer in the blood circulation.^23^

**Figure 5:**
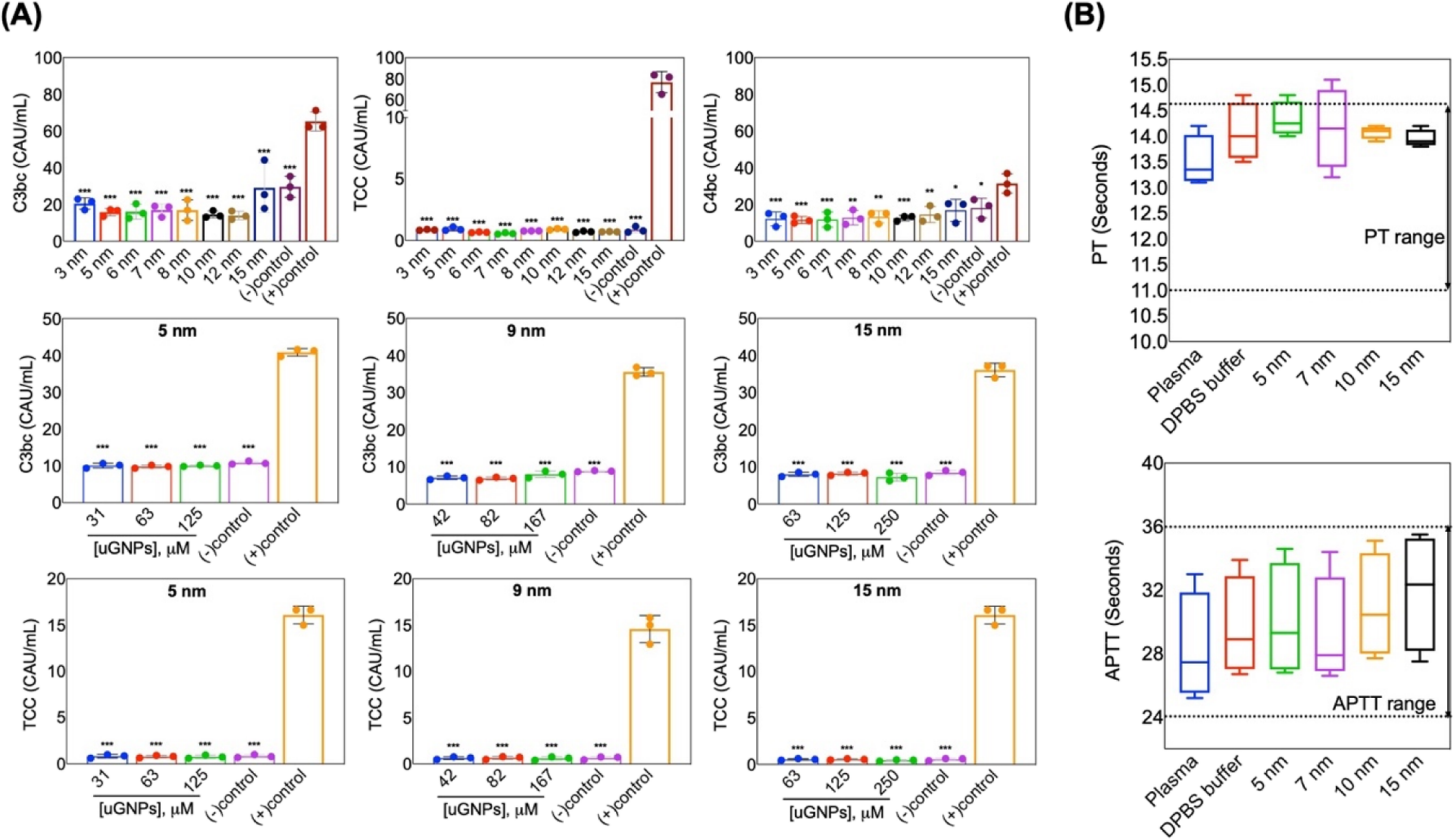
Complement activation for uGNPs by *ex vivo* incubation in human plasma. (A) Effect of uGNPs (all sizes, upper panel) on the complement activation markers C3bc, TCC, and C4bc. Effect of uGNPs (5, 10, and 15 nm) on complement activation markers (C3bc, middle panel, and TCC, lower panel) at three different concentrations. (−) Control, T30 and (+) control, zymosan. (B) Effect of uGNPs (5, 7, 10, and 15 nm) on blood coagulation; the upper panel shows the effect of uGNPs on the extrinsic pathway, that is, PT, and the lower panel shows the effect on the intrinsic pathway, that is, APTT due to the exposure of the highest concentration of uGNPs in each of their sizes (*n* = 4). Data are expressed as the mean ± SD. ***p < 0.001, **p = 0.003, and *p = 0.02 compared to the (+) control (zymosan).

To evaluate the immune safety of uGNPs, the activation-specific markers C4bc, C3bc, and sC5b-9 (or the terminal complement complex, TCC) in plasma were assessed after incubation in plasma for 30 min with or without uGNPs. The three markers represent three nodes of the complement system. C4bc is the early stage of the complement cascade and is involved only with the classical and lectin pathway.^61,62^ C3bc is the common junction of the three complement activation pathways.^61,62^ The terminal C5b-9 complement complex is the terminal product of activation.^61,62^ The complement was activated spontaneously during the incubation measured by C4bc (18 ± 5.34 CAU/mL), C3bc (29 ± 5.7 CAU/mL) (Figure 5A, upper panel), and C5b-9 (0.86 ± 0.25 CAU/mL) (Figure 5A, upper panel), but no more activation was seen by the presence of uGNPs for any size. PBS added to plasma served as the negative control, and zymosan-treated plasma served as the positive control. From Figure 5A, it can be concluded that treatment of uGNPs with any sizes did not elicit any stronger activation than the negative group (Figure S12) and caused much smaller complex activation than the positive (zymosan) group (*p* < 0.01). The complement activation levels provoked by uGNP_5_, uGNP_9_, and uGNP_15_ at various concentrations were also evaluated (Figure 5A, middle and lower panels). The result showed that for these three nanoparticles, the immunogenicity was substantially lower than that of the zymosan control within the concentrations examined.

For a successful translational application of nanoparticles into a clinical setting, it is imperative to understand their interaction with blood and its components. There have been many recent investigations of nanoparticle interacting with blood and blood components.^63−65^ Mostly, the hemolytic effect is quantitatively estimated for understanding nanoparticle interactions.^66^ Blood coagulation is another relevant test which can be extremely useful in assessing the coagulation factors involved upon exposure to nanoparticles. In this study, the influence of uGNPs on the blood coagulation was estimated using APTT and PT tests. These are the standard tests generically used for investigating the function of the intrinsic and extrinsic pathways of coagulation. APTT is generally used to assess coagulation disorders in patients with bleeding abnormalities owing to deficiencies within the intrinsic factors which lead to induced prolonged APTT. Additionally, it also reflects the effect a biomaterial can have on coagulation. It has been known that the normal physiological levels for APTT and PT were 25.1–36.5 and 9.4–12.5 s, respectively.^25^ After incubating plasma with increasing volume of uGNPs for 10 min, the solution was mixed with different reagents for testing clotting time. The results showed that there was no significant effect of uGNPs on plasma coagulation time (APTT and PT) from a low concentration to a high concentration of uGNPs for both the pathways (Figure 5B, upper and lower panels). The results indicate that uGNPs in human blood plasma are blood-compatible, and this observation further validates that the as-synthesized particles can prove to be an exceptionally valuable asset for nanomedicine translational studies.

## Conclusions

A one-pot, process-engineered synthesis of uGNP with precise size tunability at almost a single-nanometer resolution has been reported. The synthesised uGNPs are with huge colloidal stability, within the range of renal clearable sizes, and are easily functionalizable. The detailed kinetic studies of the seeding reaction using both TA and NaCit as reducing agents provided insight into the reaction mechanism to tune the particle size very narrowly. A series of spherical uGNPs with sizes ranging from 3 to 15 nm were successfully synthesized with remarkable stability at a high salt concentration. The process-engineered size tunable strategy can be extremely beneficial for scaling up with minimum batch variability. The as-synthesized uGNPs displayed striking biocompatibility and an immunocompatible profile with no toxicity toward monocytes and macrophages. The uGNPs synthesized were invisible to the complement system, suggesting that they can be used as antigen carriers in vaccines, for targeted drug delivery in cancer therapy, and as imaging contrast enhancers in translational setting. The uGNPs also showed negligible thrombogenicity, thus supporting the potential candidacy as a futuristic nanosystem that can find its way into the clinical setup.

## Experimental Section

### Synthesis of Au Seeds

Chloroauric acid, TA, potassium carbonate, and sodium citrate were purchased from Sigma-Aldrich, United Kingdom. All materials were used as received without further purification. A homemade apparatus setup, as depicted in Figure 1, was used to synthesize uGNPs of various sizes in a one-pot fashion. In a typical synthetic procedure, 25 mL of an aqueous solution containing 2.2 mM sodium citrate (NaCit, Sigma-Aldrich), 6 mM potassium carbonate (K_2_CO_3_, Sigma-Aldrich), and 0.1 mM TA (TA, Sigma-Aldrich) was brought to boil under reflux for 5 min and then kept at 80 °C under N_2_ purge and reflux. The chloroauric acid stock solution (20 mM) was added to the reaction solution using a syringe to achieve a final Au^3+^ concentration at 125 μM. The reaction mixture was then stirred for 15 min.

### Seeded Growth of uGNPs

After the color of the seed solution was persistent, 10 mL of the solution was extracted from the flask and replaced with 10 mL of 2.2 mM sodium citrate. The reaction mixture was heated up to 80 °C before adding an aliquot of chloroauric acid (20 mM). The reaction was again allowed to proceed for at least 25 min until the color of the solution stayed constant. This process was repeated several times to obtain the uGNP products with increasing diameters. The final [Au^3+^] in each synthetic round are listed in Table 1.

**Table 1:** Au^3+^ Concentration (mM) of Each Round of uGNP Synthesis

uGNP_3_	uGNP_5_	uGNP_6_	uGNP_7_	uGNP_8_	uGNP_10_	uGNP_12_	uGNP_15_
0.125	0.15	0.175	0.2	0.225	0.25	0.275	0.3

### Investigating the Role of TA and Sodium Citrate in uGNP Synthesis

To understand the role of TA and NaCit in the synthesis of uGNPs, kinetic studies were performed using the same reaction setup. One set of experiments was conducted by varying the TA concentration and keeping the NaCit concentration fixed at 2.2 mM, and in the second set of experiments, the NaCit concentration was varied at a fixed TA concentration of 0.1 mM. The reaction was recorded using a Sony Alpha A6000 camera. The red component as a fraction of the total red, blue, and green components was extracted using MATLAB’s Image Processing Toolbox. The evolution of the red component fraction as a function of time was used as the basis for the calculation of reaction rates. The reaction profiles and the rates were used to investigate and propose the underpinning reaction mechanism and determine the corresponding kinetics.

### uGNP Characterization

The hydrodynamic diameter (*D*_H_) and zeta (ζ)-potential of the as-synthesized uGNPs were characterized with DLS and ELS using a Zetasizer Nano ZS (Malvern PANalytical Products, United Kingdom) with at least 90 scans for each sample. TEM (FEI Tecnai F20) was used to confirm the size and shape of the GNPs. The particles were first subjected to ligand exchange with oleylamine and phase-transferred to hexane. It was accomplished by mixing 0.5 mL of the as-prepared uGNP solution with 0.5 mL/1.5 mM oleylamine/hexane solution. The uGNPs/hexane solutions (10 μL) were then drop-cast onto a carbon-coated grid and allowed to air-dry overnight. The particle size distributions were analyzed using ImageJ by counting at least 100 particles. The LSPR of uGNPs was measured by UV–vis spectroscopy from 210 to 1000 nm. The LSPR was determined as the absorbance peak wavelength for each sample.

### Theoretical Calculation of Particle Numbers and Surface Area

The uGNP concentration or the number of particles per unit volume was calculated based on the particle size obtained from TEM imaging using the following equation
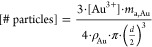
where [Au^3+^] is the molar concentration of the gold ion added in each synthesis step as summarized in Table 2, *m*_a,Au_ is the atomic mass of gold, ρ_Au_ is the density of elemental gold, and *d* is the particle size obtained with the TEM image. The total surface area of each uGNP sample is calculated according the equation
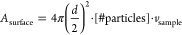


**Table 2:** DF of Each uGNP Sample to Reach the Same Surface Area

uGNPs	3 nm	5 nm	6 nm	7 nm	8 nm	10 nm	12 nm	15 nm
DF	2.18	2.42	1.86	1.47	1.27	1.22	1.02	1.00

The theoretical particle sizes of each uGNP sample were also calculated based on the #particles and the particle size of the seed (uGNP_3_) using the equation
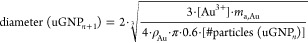


### uGNP PEGylation

The 8-arm PEG-SH (Biochempeg Scientific, Massachusetts, USA) was conjugated to uGNPs through gold-thiol linkage. The as-synthesized uGNP samples were first diluted to reach the identical surface area. The dilution factors (DFs) are summarized in Table 2. In a typical reaction, 2 mL of uGNPs was mixed with the desired amount of PEG solution and stirred for 1 h in a 60 °C water bath. The change in hydrodynamic diameter, LSPR, and the ζ-potential were measured using the same method as for bare uGNPs. The PEGylated uGNPs were purified using an Amicon centrifuge tube three times right before the next modification step.

### Flocculation Test of PEGylated uGNPs

Flocculation tests with NaCl were conducted to both bare uGNPs and PEGylated uGNPs. NaCl (1 M) was added step-wise to both as-prepared uGNPs and PEGylated samples. The samples were measured by UV–vis spectroscopy after each addition of NaCl. The shift in peak position indicates the aggregation of nanoparticles.

### Cytotoxic Study on MCF7, 293A, and SW480 Cell Lines

The effect of uGNPs on the viability of MCF-7, 293A, and SW480 was determined by the MTS assay. The MTS tetrazolium compound is bio-reduced by cells into a colored formazan product which is soluble in cell culture media. Cells were cultured in Dulbecco’s modified essential medium (DMEM, Gibco, Thermo Fisher Scientific, Waltham, Massachusetts, USA) with 10% FBS (Gibco) containing penicillin (50 IU/mL) and streptomycin (50 μg/mL) (Thermo Fisher Scientific). In all the experiments, cells were maintained at 37 °C in a humidified 5% CO_2_ incubator. MCF7, 293A, and SW480 were treated with uGNPs with eight sizes and at varying concentrations (0–125 μM). The cells were seed in 96-well plates at 10,000/well with the final volume (growth media + uGNP solution) to be 200 μL/well. The cells were then cultured for 24, 48, and 72 h, respectively. A positive control of cells with DMEM only and a negative control of cells with the DMEM/MTS assay reagent were also seeded in triplicate. 20 μL of the CelTiter 96 AQ_ueous_ One Solution (Promega, Wisconsin, USA) MTS reagent was added into each well, and the cells were then incubated at 37 °C, 5% CO_2_ for 0.5–4 h. The absorbance of each well was measured at 490 nm (650 as the reference wavelength) using a plate reader (VersaMax, Molecular Devices, California, USA). All experiments were conducted in triplicate. The percentage viability of cells was calculated according to the following equation: cell viability (%) = ((absorbance of treated cells/absorbance of control cells) × 100). Blank = no cell, only media with the MTS reagent.

### Compatibility Test of uGNPs to the Acute Monocyte Leukaemia Cell Line, THP1

The cell lines were maintained in the RPMI 1640 medium (Gibco), supplemented with 10% FBS (Gibco), 1% penicillin–streptomycin (Thermo Fisher Scientific), 1% l-glutamate, and 1 mM sodium pyruvate (Gibco). THP-1 cells were cultured in a humidified chamber at 37 °C, 5% CO_2_. The MTS assay as described previously was performed to evaluate the cytotoxicity of uGNPs on the THP-1 cell line. Briefly, cells (100,000 cells/mL) were seeded into a 96-well plate in triplicate and incubated for 72 h (37 °C, 5% CO_2_) with varying concentrations of uGNPs (0–125 μM). A positive control of THP-1 with RPMI only and a negative control with the RPMI/MTS assay reagent were also seeded in triplicate. After the incubation period with the particles, the MTS assay was performed and the cell viability (%) was determined as described previously. Simultaneously, the membrane integrity assay using the CytoTox-ONE homogeneous reagent (Promega) was also carried out on the THP-1 differentiated macrophage induced by PMA, 100 nM (Sigma-Aldrich, Saint Luis, MO, USA) for 48 h. After this differentiation window, the cells were replenished with the fresh complete RPMI medium without PMA for additional 48 h to allow for cell recovery. Cell differentiation was determined by evaluating cell adhesion and spreading using a bright-field microscope (Axio, Carl Zeiss AG, Oberkochen, Germany). The differentiated macrophage was incubated for 72 h (37 °C, 5% CO_2_) with varying concentrations of uGNPs (0–125 μM). In a nutshell, the assay is a rapid fluorescence measurement of the release of LDH from cells with a compromised membrane. Estimation of the leakage of components from the cytoplasm into the surrounding cell culture medium has been accepted as a valid method to determine the number of non-viable/compromised cells. As a positive control for maximum LDH release, 2 μL of the lysis solution (provided in the kit) was added to the untreated or control wells, followed by the addition of 100 μL of the CytoTox-ONE homogeneous reagent to all the wells. The plate was then incubated at 22 °C for 10 min; this step was followed by the addition of 50 μL of the stop solution (provided in the kit), and the plate was shaken for 10 s. The final step involves the fluorescence measurement at an excitation wavelength and an emission wavelength of 560 and 590 nm, respectively, with a Spark 10M multimode microplate reader (TECAN, Switzerland).

### Cytokine Analysis in THP-1 and PMA-Induced THP-1 Differentiated Macrophages

Differentiated (macrophage-like) and undifferentiated (monocyte-like) THP-1 cells (1 × 10^5^ cells/mL) were treated with increasing concentrations of uGNPs with and without lipopolysaccharide (LPS, 10 ng/mL) for 24 h. Addition of LPS is primarily used for the following: (1) as a positive control and (2) to mimic endotoxin contamination which leads to pro-inflammatory responses. At the end of the uGNP treatment window, 1 mL of cells from individual wells was aliquoted and centrifuged at 1000 rpm for 5 min. The supernatant was collected and kept at −80 °C for cytokine analysis. The meso scale discovery (MSD) multiplex assay platform was used here to allow quantitation of multiple cytokines in the same sample. THP-1 monocytic and macrophage-like cell culture media were collected, and the corresponding cytokine levels were measured using the ultrasensitive electrochemical luminescence immunoassay on the MSD assay platform. The luminescence readout was performed on the MesoScale Diagnostics Sector Imager 6000. All reagents and calibrators will be supplied by MesoScale Discovery, and the samples were analyzed at the Core Biochemical Assay Laboratory (NHS Cambridge University Hospitals; UK).

### Complement Activation of uGNPs by *In Vivo* Plasma Incubation

To prepare plasma, whole blood was collected from six healthy donors in 4.5 mL sterile tubes (Nunc, Roskilde, Denmark) containing the thrombin inhibitor lepirudin (Refludan; Pharmion ApS, Copenhagen, Denmark) at a final concentration of 50 μg/mL to prevent blood clotting.^67^ Blood was immediately centrifuged at 3000*g* for 15 min at 4 °C to obtain plasma, which was pooled. Complement activation was studied by incubating 100 μL of pooled plasma with uGNPs for 30 min at 37 °C in 1.8 mL round-bottom sterile polypropylene Nunc cryotubes (Nunc). After incubation, activation was stopped by adding ethylenediaminetetraacetic acid (20 mM) and the plasma was stored at −80 °C until further analysis. Activation markers at the level of C4 (C4bc), C3 (C3bc), and the soluble C5b-9 (sC5b-9) TCC were analyzed in the plasma samples using ELISA as described previously.^68^ Briefly, the assays were based on monoclonal antibodies detecting neoepitopes exposed after activation, hence specifically measuring only activation-specific fragments (C4bc and C3bc) and complexes (sC5b-9).

### Coagulation Test

Blood was collected from healthy individuals with their due consent and then mixed with the anticoagulant (3.2% NaCit) in a 9:1 ratio (by volume). To obtain the platelet-poor plasma, blood was centrifuged at 1000*g* for 15 min at room temperature. The plasma obtained after centrifugation was then incubated with uGNPs for 10 min at 37 °C in a water bath. The samples were then taken for coagulation analysis, that is, APTT to study the intrinsic pathway and PT for the extrinsic pathway using a Stago Compact Max (USA), an automatic coagulation analyzer. For the control sets, Dulbecco’s PBS was used as a volume control and unfractionated heparin (1000 U/mL, Sigma-Aldrich) was used as negative control for the APTT experiment. Briefly, in the Stago Compact Max, to evaluate the PT value, each 50 μL of the sample (uGNP-treated or control) was taken automatically to the reaction chamber (cuvette), followed by addition of 100 μL of the thromboplastin (STA Neoplastic R15) phospholipid and then 50 μL of CaCl_2_ (0.025 M). Within 3–5 s, the PT value was measured and shown in the machine. The normal range of PT is 11–16 s, which depends on the analytical technique and source of thromboplastin. According to the Stago Compact Max, the standard PT is between 11 and 14.5 s. To measure the APTT, 50 μL of the sample was taken to the reaction chamber (cuvette), same as mentioned for the PT measurement. Each sample was then treated with 50 μL of the phospholipid, the kaolin activator (STA C.K. Prest 5), and then last, 50 μL of CaCl_2_ (0.025 M). The APTT value was then measured. The time required for this measurement was ∼3–5 s. The normal range of APTT is 24–37 s. According to the Stago Compact Max, the standard time for APTT is from 24 to 37 s (Table 3). Additionally, absorbance and fluorescence spectra of uGNPs were recorded in a Thermo Scientific Evolution 300 instrument from 200 to 700 nm. The scan speed was 400 nm/min using a bandwidth of 1.0 nm. Briefly, blood plasma was isolated from healthy volunteers and incubated with the increase of uGNPs for 10 min, and the spectra were obtained. Similarly, the fluorescence spectra of plasma incubated with uGNPs for 10 min were recorded in a QuantaMaster 8000 Spectrofluorometer (HORIBA Europe GmbH, Gothenburg, Sweden). For the fluorescence scan, the excitation wavelength was 285 nm and the emission range was from 305 to 550 nm with 0.5 nm of slit width for both the excitation and emission apertures.

**Table 3:** Coagulation Test of uGNPs

control 1	control 2	control 3	set 1	set 2	set 3	set 4
1 mL plasma	999 μL plasma + 1 μL heparin	900 μL plasma + 100 μL PBS	900 μL plasma +15 μM uGNP_5_	900 μL plasma + 20 μM uGNP_7_	900 μL plasma + 25 μM uGNP_10_	900 μL plasma + 30 μM uGNP_15_

### Principal Component Analysis

PCA was performed on the uGNPs and pro-inflammatory cytokine data using R (v3.5.3) and FactomineR^69^ and factoextra packages.^70^ To compute the quantitative variables and factors, the PCA was calculated by normalizing the same weight from the first eigenvalue of the same variance. The representation of the variables was used to describe the dimensions in the plot.

### Statistical Analysis

All the experiments were conducted independently twice with different batches of nanoparticles, and the subsequent analysis was performed in triplicate. Statistical analysis was performed using ANOVA in GraphPad Prism 8.0 (GraphPad Software, San Diego, USA), followed by the *t*-test with Bonferroni correction for comparison with the untreated/control group. Statistical significance was determined at a *p*-value < 0.05.

## Supporting Information Available

Kinetics study, uGNP characterization, uGNP stability, uGNP surface PEGylation, flocculation test, uGNP cytotoxicity, and complement activation (PDF)
