# Supplementary material for: Innate Immune Invisible Ultrasmall Gold Nanoparticles—Framework for Synthesis and Evaluation
Source: ACS Appl Mater Interfaces. 2021 May 12;13(20):23410–22. doi: 10.1021/acsami.1c02834 (PMC8289183; doi:10.1021/acsami.1c02834)
Supplement: Supplementary file 1 — am1c02834_si_001.pdf [file am1c02834_si_001.pdf]

Supporting Information

Innate immune invisible ultra -small gold nanoparticles - Framework for synthesis and evaluation

*Geyunjian Harry Zhu[#,1], Mohammad Azharuddin[#,2], Rakibul Islam[3], Hassan Rahmoune[1], Suryyani Deb[4], Upasona Kanji[4], Jyotirmoy Das[2], Johannes Osterrieth[1], Parminder Aulakh[5], Hashi Ibrahim-Hashi[1], Raghav Manchanda[1], Per H Nilsson[3,6], Tom Eirik Mollnes[3,7], Maitreyee Bhattacharyya[8], Mohammad M. Islam[9], Jorma Hinkula[2], Nigel K H Slater[1], and Hirak K Patra[#, *,1, 10]*

[1]Dept of Chemical Engineering and Biotechnology, University of Cambridge, Cambridge, UK

[2]Dept of Biomedical and Clinical Sciences (BKV), Linkoping University, Linkoping, Sweden

[3]Dept of Immunology, Oslo University Hospital, University of Oslo, Oslo, Norway

[4]Dept of Biotechnology, Maulana Abul Kalam Azad University of Technology (MAKAUT), India

[5]Institute of Manufacturing, University of Cambridge, Cambridge, UK

[6]Linnaeus Center for Biomaterials Chemistry, Linnaeus University, Kalmar, Sweden

[7]Research Laboratory, Nordland Hospital, Bodø, and Faculty of Health Sciences, K.G. Jebsen TREC, University of Tromsø, Norway

[8]Institute of Haematology and Transfusion medicine, Calcutta Medical College, Calcutta, India

[9]Massachusetts Eye and Ear and Schepens Eye Research Institute, Dept of Ophthalmology, Harvard Medical School, Boston, USA

[10]Department of Surgical Biotechnology, University College London (UCL), London, UK

*Correspondence: Hirak K Patra (hirak.patra@ucl.ac.uk)

Kinetics study

A red component analogue was used to track the progression of the reaction as the red colour is indicative of the formation of uGNPs. This technique was employed as a substitute to in-situ UV-Vis as this was not possible due to the high reaction temperature. The change in red component as a fraction of the total red, blue and green component of a particular image segment was determined using MATLAB's Image Processing Toolbox™. A robust version of local regression using weighted linear least squares and a 2nd degree polynomial model ('rloess') was employed to smooth the data. The recording was captured at 24.3-megapixel resolution by a Sony Alpha A6000 (Tokyo, Japan).

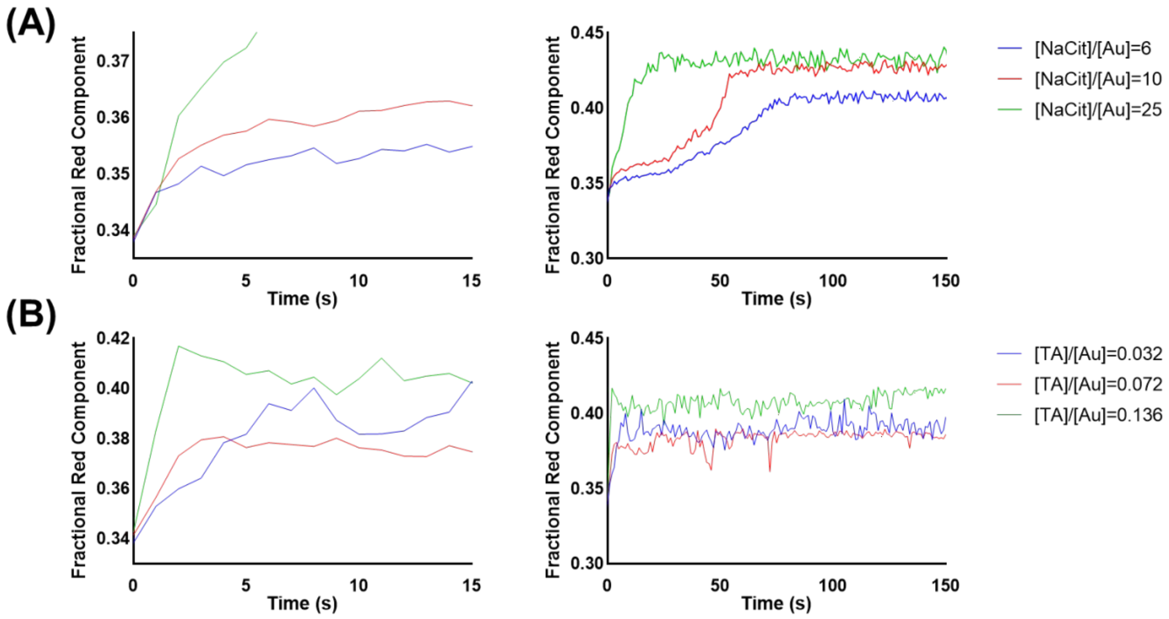

Figure S1. Fractional red component as functions of time for different ratios of (A) NaCit/Au (B) TA/Au

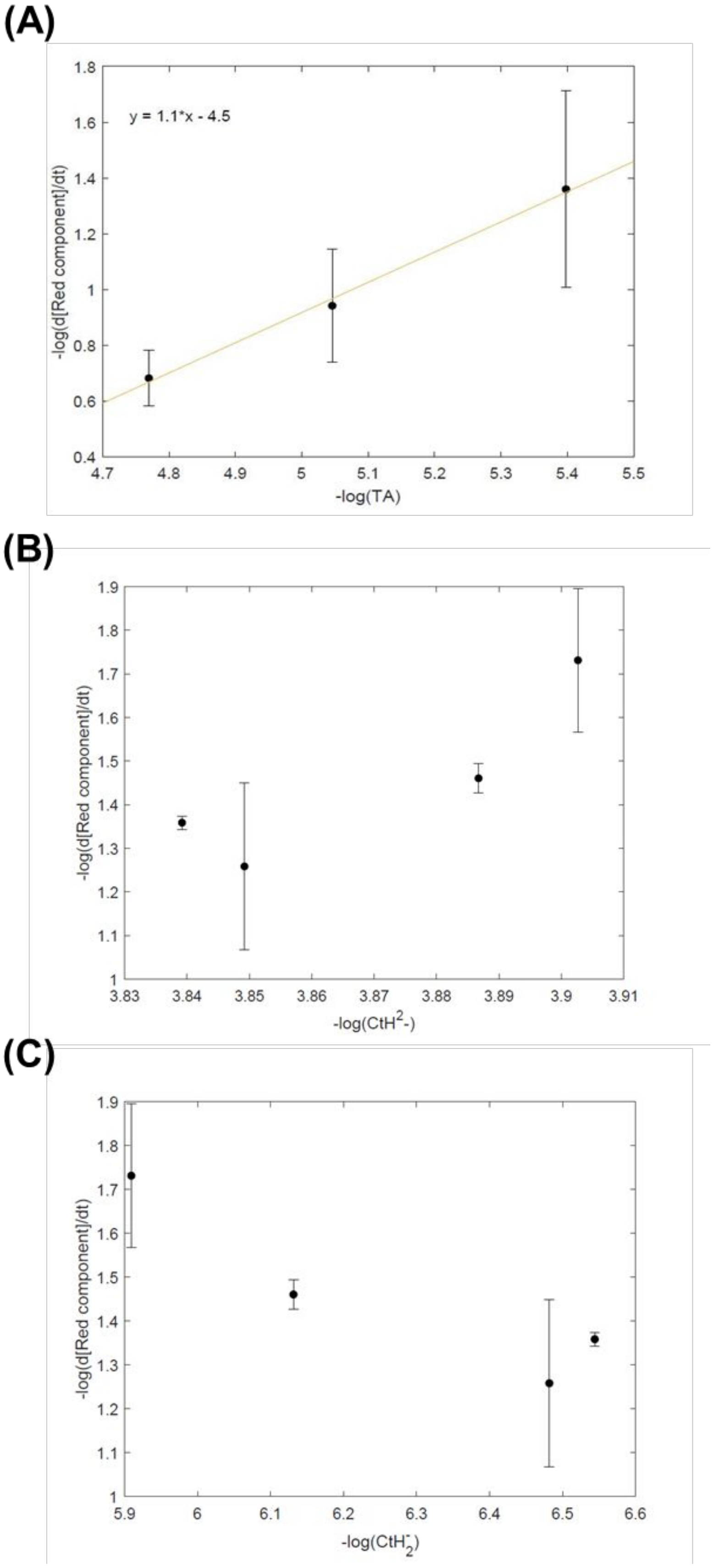

Figure S2. Log-log plot correlating TA (A), CtH^{2-}(B), CtH_2^-(C) concentration to the d(Red component)/dt

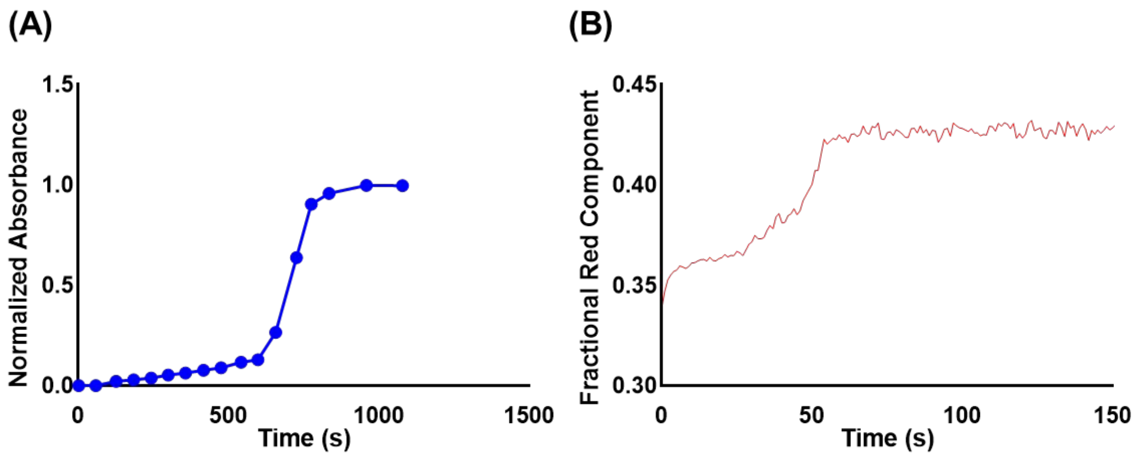

Figure S3. (A) Turkevich reaction profile replot from[1] *(B) Mühlpfordt reaction profile*

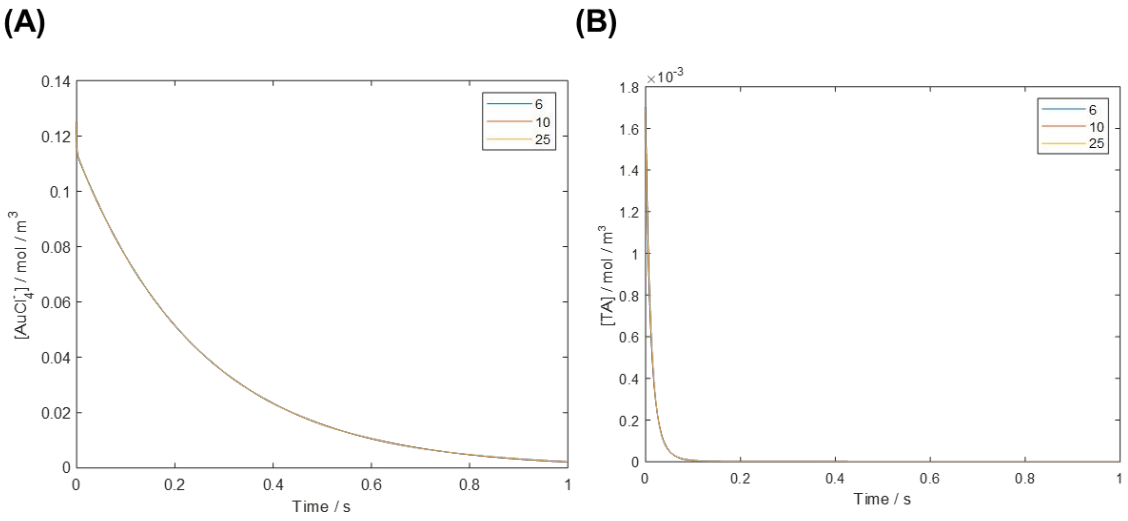

Figure S4. Simulated concentration profiles of (A) AuCl4- (B) TA at different NaCit/Au ratios

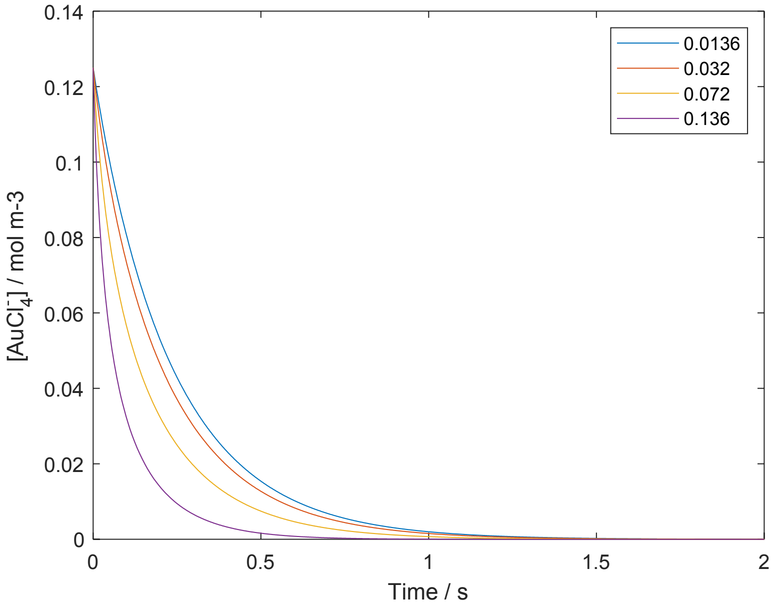

Figure S5. Simulated concentration profile of $AuCl_4^-$ at different TA/Au ratios

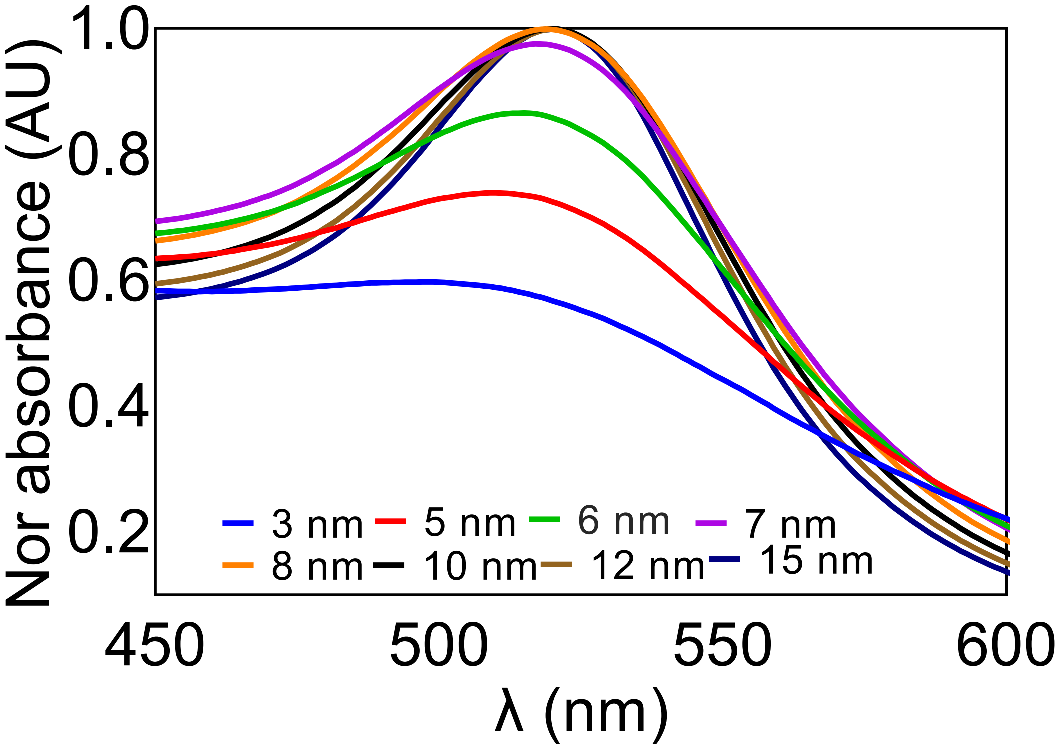

Figure S6. UV-VIS spectra of $uGNP_3$ to $uGNP_{15}$

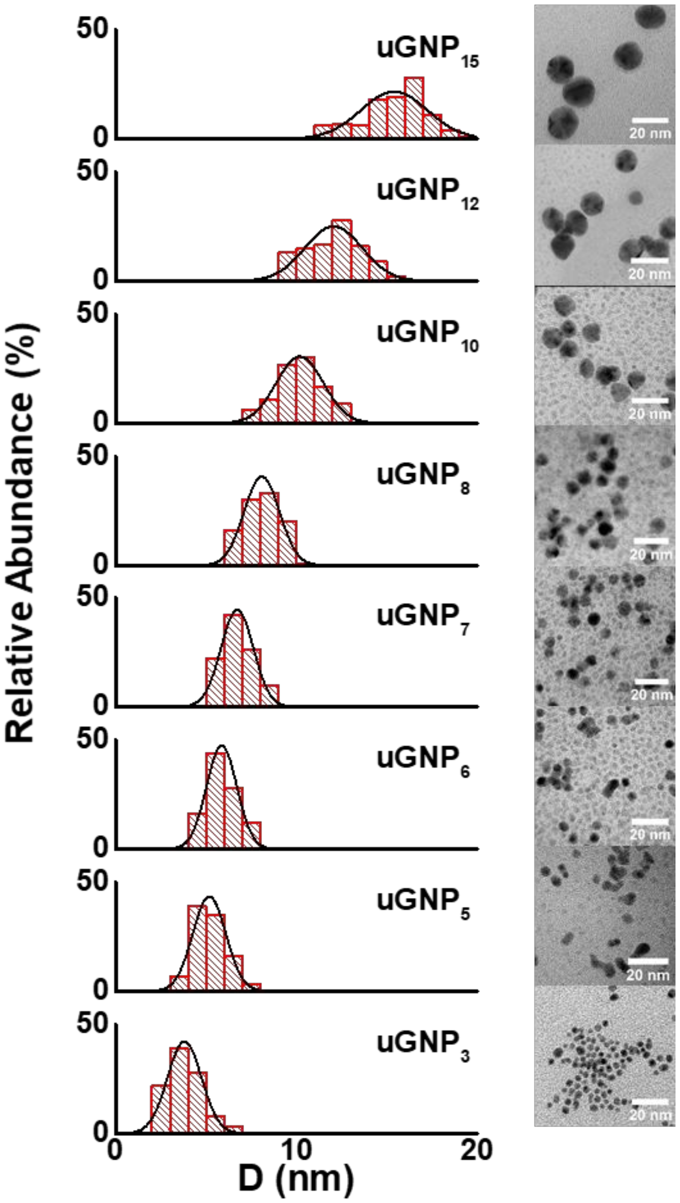

Figure S7. Size distribution and respective TEM images for uGNPs

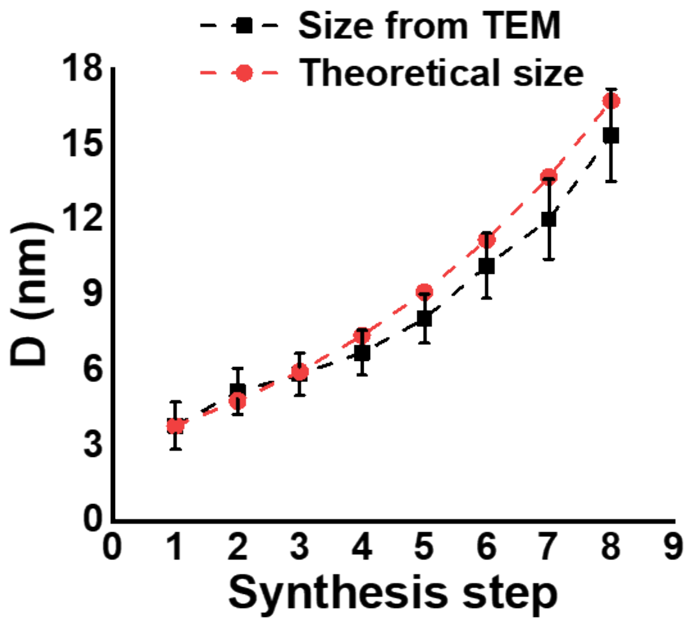

Figure S8. Theoretical and actual sizes of uGNPs

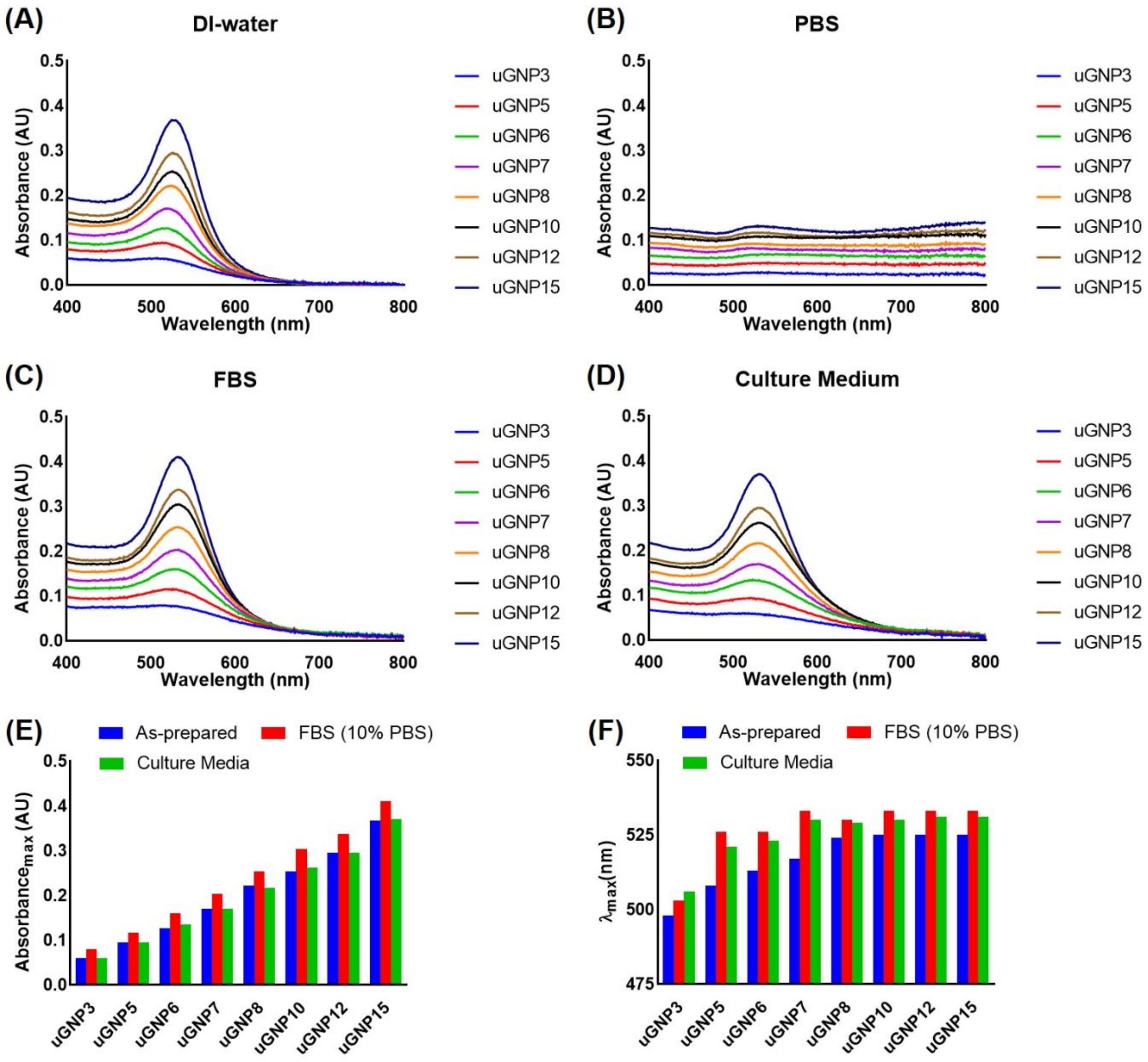

Figure S9. Stability of uGNPs in various dispersing media. UV-Vis spectra of uGNPs dispersed in (A) DI-water, (B) PBS (1X, pH 7.4), (C) fetal bovine serum (10% in PBS), and (D) colourless Dulbecco's Modified Eagle Medium supplemented with 10% FBS. Summary of (E) maximum absorbance and (F) λ_{max} in DI-water, FBS solution, and culture medium. Measurements were performed after 24 hr of incubation.

The stability of citrate capped uGNPs in various dispersing media was studied. As-prepared uGNPs were dispersed in DI-water, PBS (1X, pH 7.4), foetal bovine serum (FBS, 10% in PBS) solution, and culture medium at 1:2 volume ratio for 24 hours and the respective UV-Vis spectra were recorded. It can be seen that uGNPs became colloidally unstable in PBS as evidence by the complete flattening of LSRP peak. Surprisingly the uGNPs stayed relatively stable in FBS and culture medium as the overall shape of UV-Vis spectra remained unchanged. The absorbance of uGNPs increased in FBS solution and remained at the same level in culture

medium, as compared with DI-water. In terms of shift in λ_{max}, upshifts in peak position were observed with uGNPs dispersed in both FBS and culture medium. Larger degree of shifts was resulted in FBS than in culture medium. Changes in peak positions were more prominent for intermediate sizes ($uGNP_5$ to $uGNP_8$).

In terms of surface chemistry, it is widely acknowledged that antifouling materials such as polyethylene glycol (PEG) and zwitterionic coatings can provide the stealth effect to reduce or alter protein adsorption onto nanoparticle surface leading to longer circulating time and improved targeting efficiency.[2,3]

To further prove the functionalization with PEG and to examine the increased colloidal stability of PEGylated uGNPs in high ionic strength solution (mimicking physiological condition), flocculation test was carried out. The PEGylated uGNPs were initially suspended in aqueous media, and aliquots of 1M NaCl were added into the nanoparticle solution. After thorough mixing, the LSPR was measured by UV-Vis spectroscopy (Figure 2B). The shift of absorbance peak position (λmax) to larger wavelength indicated aggregation of uGNPs. Both bare uGNPs and uGNP-PEG demonstrated good colloidal stability up to ~50 mM of NaCl, but the λmax of bare uGNPs shifts to larger wavelength ionic strength of the suspension increased to close 100 mM. The only exception is $uGNP_3$ which exhibited extraordinarily high colloidal stability. This was likely due to the extremely small size of uGNP3. As the size of the uGNPs increased, the shift of λmax became larger and larger, indicating less stability. In contrast, uGNP-PEG-SH with all sizes did not show any shift in λmax. The result shows that the process engineered uGNPs are easily modified with simple gold-thiol chemistry and stabilised with PEG coating.

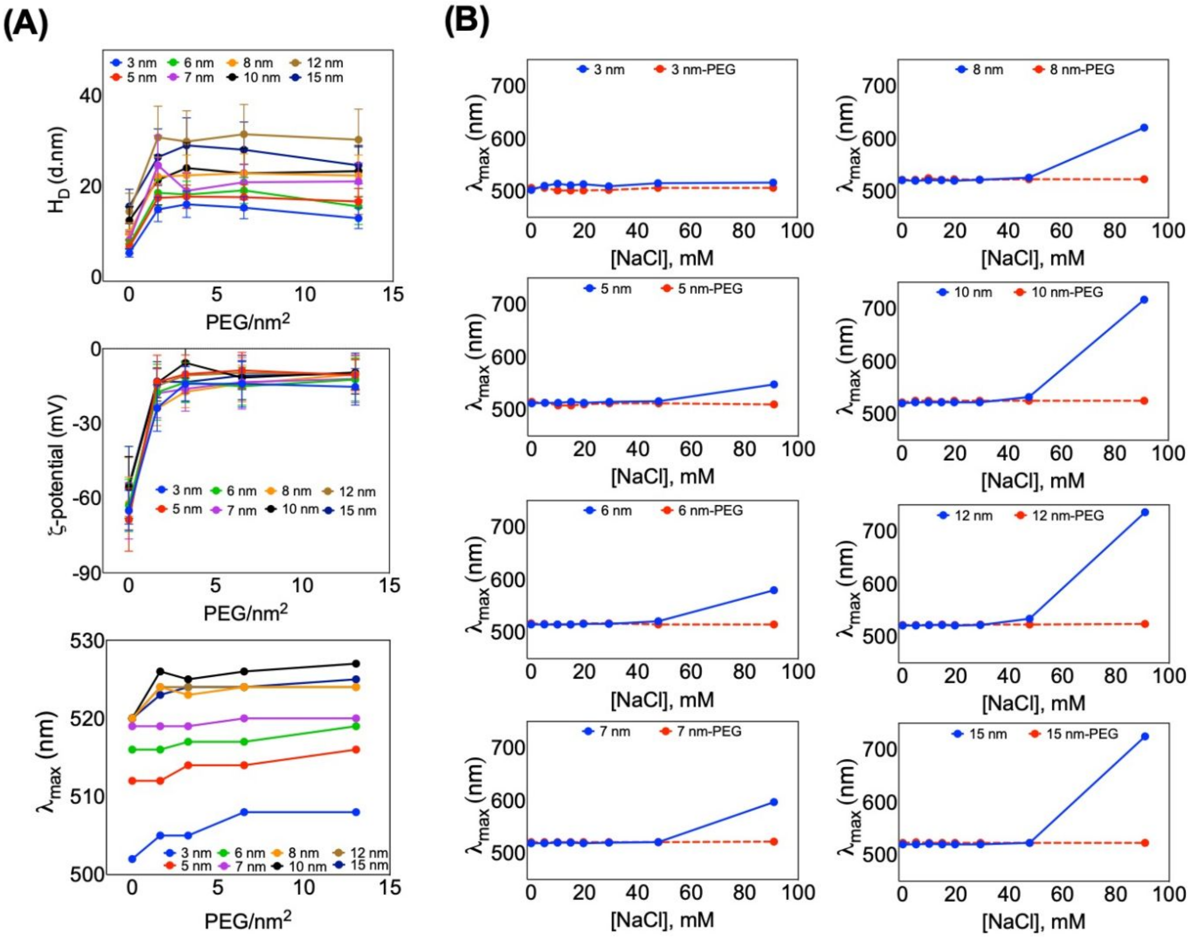

Figure S10. (A) Changes in H_D, ζ-potentials, and λ_{max} of uGNPs functionalised with various amount of 8-arm PEG; (B) flocculation experiments of bare uGNPs and PEG-uGNPs as shown by λ_{max} changes with addition of NaCl.

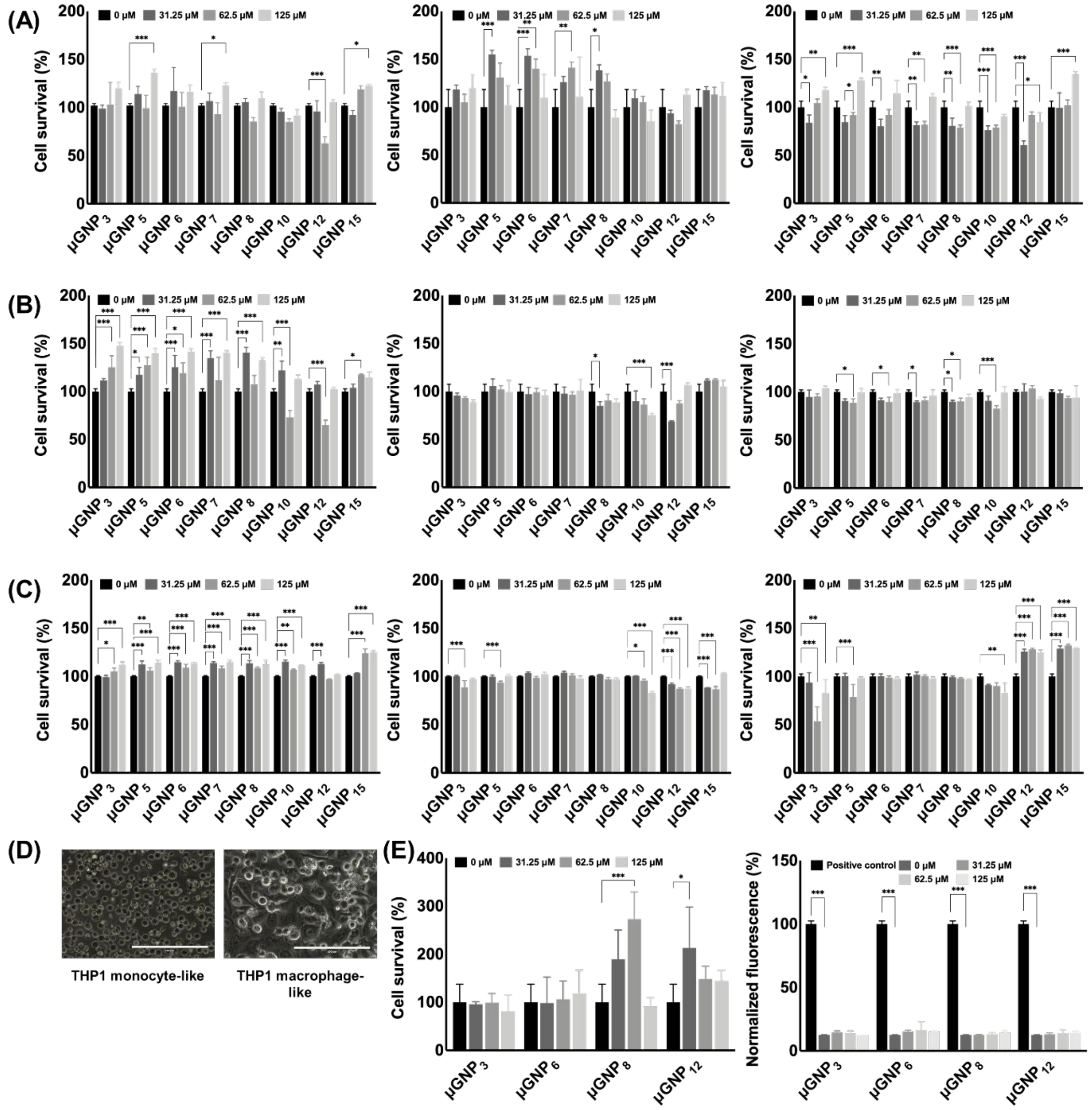

Figure S11. MTS assays with MCF7 (A), SW480 (B), and 293A (C) cell lines at 24, 48, and 72 hours. (D) Microscopic images of THP1 monocyte-like and THP1 macrophage-like. (E) Cytotoxicity of uGNPs to THP1 monocyte-like as evaluated by MTS (left) and to THP1 macrophage-like as evaluated by LDH (right) assays.

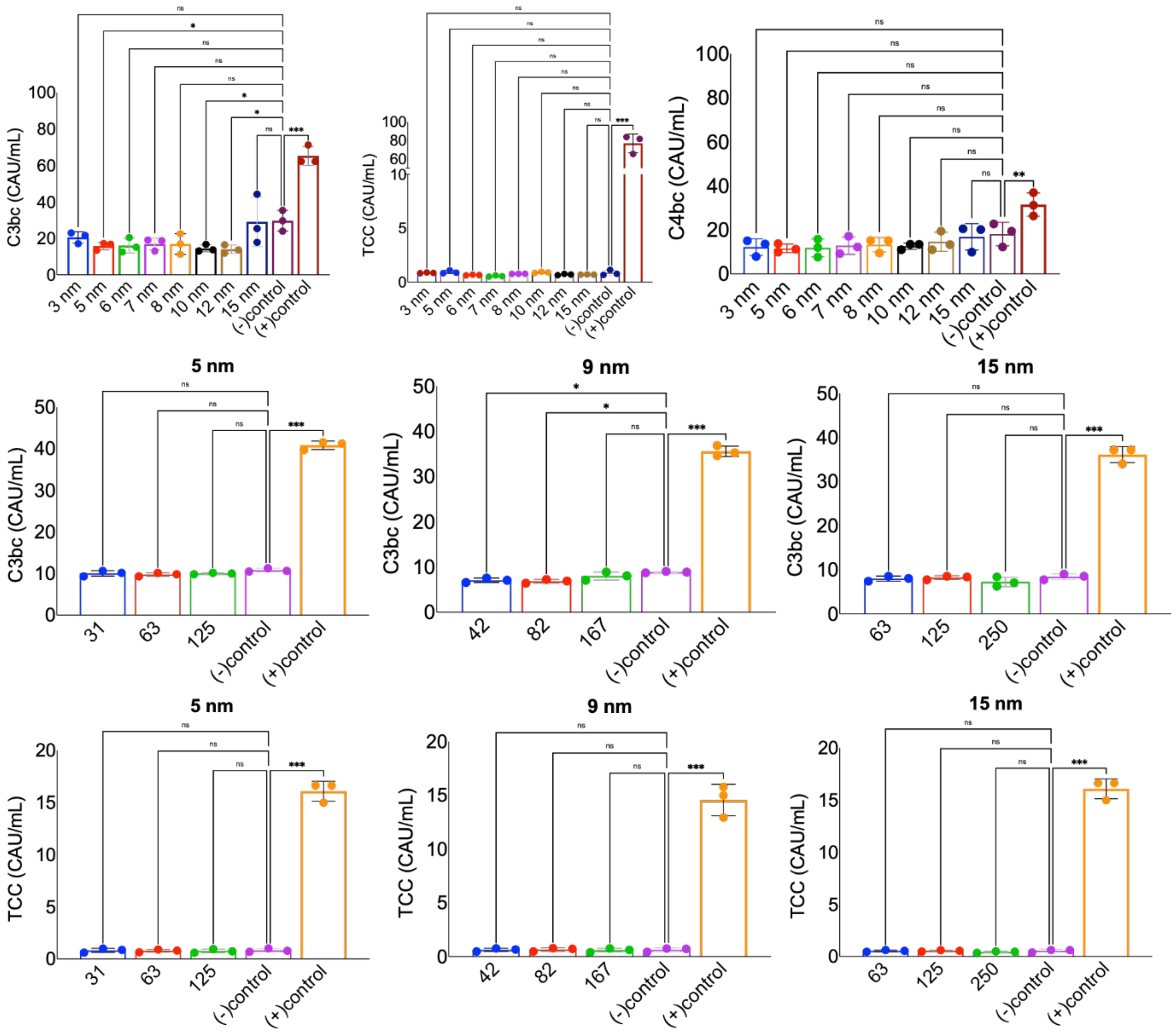

*Figure S12. Complement activation for uGNPs by ex vivo incubation in human plasma. Effect of uGNPs (all sizes, upper panel) on complement activation markers C3bc, TCC, and C4bc respectively. Effect of uGNPs (5,9, and 15 nm) on complement activation marker (C3bc, middle panel and TCC, lower panel) at three different concentration. (-) control, T30 and (+) control, Zymosan. Data is expressed as mean ± SD. ***p < 0.001, **p=0.006 and *p= < 0.1 compared to the (-) control (PBS).*

References

(1) Agunloye, E.; Panariello, L.; Gavriilidis, A.; Mazzei, L. A Model for the Formation of Gold Nanoparticles in the Citrate Synthesis Method. *Chem. Eng. Sci.* **2018**, *191*, 318–331.

(2) Pelaz, B.; del Pino, P.; Maffre, P.; Hartmann, R.; Gallego, M.; Rivera-Fernández, S.; de la Fuente, J. M.; Nienhaus, G. U.; Parak, W. J. Surface Functionalization of Nanoparticles with Polyethylene Glycol: Effects on Protein Adsorption and Cellular Uptake. *ACS Nano* **2015**, *9*, 6996–7008.

(3) Moyano, D. F.; Saha, K.; Prakash, G.; Yan, B.; Kong, H.; Yazdani, M.; Rotello, V. M. Fabrication of Corona-Free Nanoparticles with Tunable Hydrophobicity. *ACS Nano* **2014**, *8*, 6748–6755.
